# Electron microscopy holdings of the Protein Data Bank: the impact of the resolution revolution, new validation tools, and implications for the future

**DOI:** 10.1007/s12551-022-01013-w

**Published:** 2022-12-02

**Authors:** Stephen K. Burley, Helen M. Berman, Wah Chiu, Wei Dai, Justin W. Flatt, Brian P. Hudson, Jason T. Kaelber, Sagar D. Khare, Arkadiusz W. Kulczyk, Catherine L. Lawson, Grigore D. Pintilie, Andrej Sali, Brinda Vallat, John D. Westbrook, Jasmine Y. Young, Christine Zardecki

**Affiliations:** 1 Research Collaboratory for Structural Bioinformatics Protein Data Bank, Rutgers, The State University of New Jersey, Piscataway, NJ 08854 USA; 2 Institute for Quantitative Biomedicine, Rutgers, The State University of New Jersey, Piscataway, NJ 08854 USA; 3 Cancer Institute of New Jersey, Rutgers, The State University of New Jersey, New Brunswick, NJ 08901 USA; 4 Research Collaboratory for Structural Bioinformatics Protein Data Bank, San Diego Supercomputer Center, University of California San Diego, La Jolla, CA 92093 USA; 5 Department of Chemistry and Chemical Biology, Rutgers, The State University of New Jersey, 174 Frelinghuysen Road, Piscataway, NJ 08854 USA; 6 Department of Bioengineering, Stanford University, Stanford, CA USA; 7 Division of CryoEM and Bioimaging, SSRL, SLAC National Accelerator Laboratory, Stanford University, Menlo Park, CA USA; 8 Department of Cell Biology and Neuroscience, Rutgers, The State University of New Jersey, Piscataway, NJ 08854 USA; 9 Department of Biochemistry and Microbiology, Rutgers, The State University of New Jersey, Piscataway, NJ 08901 USA; 10 Research Collaboratory for Structural Bioinformatics Protein Data Bank, Department of Bioengineering and Therapeutic Sciences, Department of Pharmaceutical Chemistry, Quantitative Biosciences Institute, University of California San Francisco, San Francisco, CA 94158 USA

**Keywords:** Electron microscopy, Protein Data Bank, PDB, PDB-Dev, Electron Microscopy Data Bank, EMDB, Electron microscopy data resource, Resolution Revolution, Cryo-electron microscopy, Cryo-electron tomography, Sub-tomogram averaging, Electron crystallography, Micro-electron diffraction, Icosahedral viruses, Ribosomes, Integral membrane proteins, SARS-CoV-2 spike proteins, Integrative or hybrid methods, Structure validation, Q-score

## Abstract

As a discipline, structural biology has been transformed by the three-dimensional electron microscopy (3DEM) “Resolution Revolution” made possible by convergence of robust cryo-preservation of vitrified biological materials, sample handling systems, and measurement stages operating a liquid nitrogen temperature, improvements in electron optics that preserve phase information at the atomic level, direct electron detectors (DEDs), high-speed computing with graphics processing units, and rapid advances in data acquisition and processing software. 3DEM structure information (atomic coordinates and related metadata) are archived in the open-access Protein Data Bank (PDB), which currently holds more than 11,000 3DEM structures of proteins and nucleic acids, and their complexes with one another and small-molecule ligands (~ 6% of the archive). Underlying experimental data (3DEM density maps and related metadata) are stored in the Electron Microscopy Data Bank (EMDB), which currently holds more than 21,000 3DEM density maps. After describing the history of the PDB and the Worldwide Protein Data Bank (wwPDB) partnership, which jointly manages both the PDB and EMDB archives, this review examines the origins of the resolution revolution and analyzes its impact on structural biology viewed through the lens of PDB holdings. Six areas of focus exemplifying the impact of 3DEM across the biosciences are discussed in detail (icosahedral viruses, ribosomes, integral membrane proteins, SARS-CoV-2 spike proteins, cryogenic electron tomography, and integrative structure determination combining 3DEM with complementary biophysical measurement techniques), followed by a review of 3DEM structure validation by the wwPDB that underscores the importance of community engagement.

## Introduction

The Protein Data Bank (PDB) was the first open-access digital data resource in biology (Berman 2008). Now in its 51st year of continuous operations, it was established in 1971 with just seven protein structures (Protein Data Bank 1971; Burley et al. 2022c). As of mid-2022, the PDB archive housed > 190,000 3D structures of proteins and nucleic acids (DNA and RNA) and their complexes with one another and with small-molecule ligands (e.g., enzyme co-factors, drugs). Throughout its history, the PDB has been regarded as a pioneer in the open-access data movement. Nearly 60,000 structural biologists working on every permanently inhabited continent have generously deposited 3D structure information (atomic coordinates, experimental data, and related metadata) to the archive over more than 50 years. Currently supported experimental methods include macromolecular crystallography (MX), nuclear magnetic resonance spectroscopy (NMR), 3D electron microscopy (3DEM), Electron Crystallography (EC), and micro-electron diffraction (micro-ED). Today, millions of PDB data consumers worldwide working in fundamental biology, biomedicine, bioengineering, biotechnology, and energy sciences enjoy no-cost access to 3D biostructure information with no limitations on data usage.

Since 2003, the PDB archive has been jointly managed by the Worldwide Protein Data Bank (wwPDB, wwpdb.org) partnership (Berman et al. 2003; wwPDB consortium 2019). wwPDB Full Members include the US-funded Research Collaboratory for Structural Bioinformatics Protein Data Bank (RCSB PDB, RCSB.org, (Berman et al. 2000; Burley et al. 2021; Burley et al. 2022c; Burley et al. 2022b)); the Protein Data Bank in Europe (PDBe, PDBe.org, (Armstrong et al. 2020)); Protein Data Bank Japan (PDBj, PDBj.org, (Bekker et al. 2022)); the Electron Microscopy Data Bank (EMDB, emdb-empiar.org, (Tagari et al. 2002; Lawson et al. 2016)); and the Biological Magnetic Resonance Bank (BMRB, bmrb.io, (Ulrich et al. 2008)). Protein Data Bank China (PDBc) was recently admitted to the wwPDB as an Associate Member. wwPDB partners are committed to the FAIR (Findability, Accessibility, Interoperability, and Reusability) (Wilkinson et al. 2016) and FACT (Fairness, Accuracy, Confidentiality, and Transparency) (van der Aalst et al. 2017) Principles emblematic of responsible data stewardship in the modern era.

The RCSB PDB is headquartered at Rutgers, The State University of New Jersey with additional performance sites at the University of California San Diego and the University of California San Francisco. Within the wwPDB, RCSB PDB serves as the designated PDB Archive Keeper, responsible for archiving ~ 100 TB of digital information and a physical archive that includes correspondence and other artifacts accumulated since the early 1970s. Based on a conservative estimate of US$100,000 for the replacement cost of an individual PDB structure, replacement of the entire archive would cost about US$20 billion (Sullivan et al. 2017).

This review article, published in a special issue of *Biophysical Reviews* honoring Professor Haruki Nakamura on the occasion of his 70th birthday, is focused on 3DEM structures archived within the PDB and the impact of the 3DEM “Resolution Revolution” (Kuhlbrandt 2014; Herzik 2020) on basic and applied research across fundamental biology, biomedicine, energy sciences, and bioengineering and biotechnology. Nakamura served as founding Director of Protein Data Bank Japan (PDBj) from 2000 to 2017. In 2003, he was one of the co-founders of the wwPDB partnership (Berman et al. 2012; Berman et al. 2003). Under Nakamura’s leadership, PDBj assumed PDB data-in responsibility for all structure depositions coming from Asia and the Middle East. PDBj data-out activities play a unique role within the wwPDB partnership, delivering information from the PDB archive on its PDBj.org website in English, Japanese, Korean, and Mandarin (both Traditional and Modern) (Kinjo et al. 2018, 2017, 2012, 2010).

## Growth, resolution, and composition/complexity of 3DEM structures in PDB

**Fig. 1:**
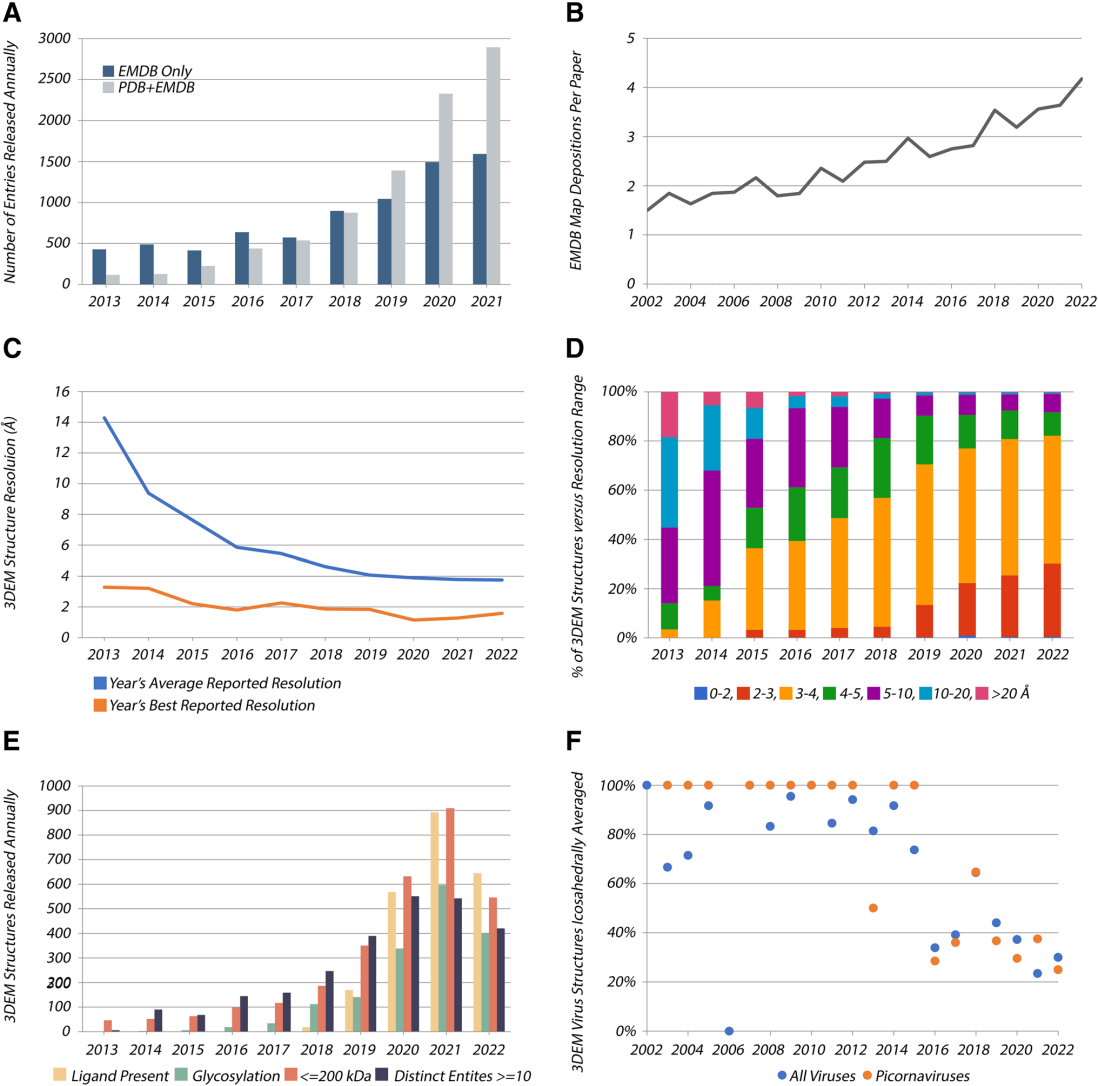
Selected annual metrics for 3DEM structures in PDB and density maps in EMDB. **A** 3DEM structures (PDB and EMDB) and density maps (EMDB only) versus time. **B** Average number of 3DEM density map depositions reported per primary publication versus time. **C** Average reported resolution (blue) and best reported resolution (orange) for 3DEM structures versus time. **D** Percentage of 3DEM structures versus resolution range versus time. **E** PDB 3DEM structures wherein ligands are present; glycosylation is evident; size of the sample macromolecule or macromolecular complex is $\le$ 200,000 Da; and the number of distinct molecular entities comprising the sample is $\ge$ 10. **F** Percentage of 3DEM virus structure depositions to PDB relying on icosahedral averaging versus time

Transformation of cryogenic electron microscopy (cryo-EM) into a mainstream structural biology technique is evidenced by (i) dramatic growth in the number of 3DEM structures over the past decade (Fig. 1A), and (ii) award of the 2017 Nobel Prize in Chemistry to Jacques Dubochet, Joachim Frank, and Richard Henderson. Deposition of atomic coordinates to the PDB and experimental density maps to EMDB is mandatory for publication of newly determined 3DEM structures in all major scientific journals. Prior to 2013, the field was limited to a small number of expert laboratories. Structure determinations were hampered by methodological bottlenecks, and only 385 3DEM structures were deposited to the PDB in this era. This paucity of structures stands in stark contrast to the present. As of mid-2022, PDB archive holdings included a total of 11,309 3DEM structures determined using single-particle cryo-EM methods, cryogenic electron tomography (cryo-ET), EC, and microED.

Single-particle Cryo-EM has become one of the most sought-after experimental methods in structural biology thanks to a host of technical advances, including modern electron microscopes (Weis and Hagen 2020), DEDs (Campbell et al. 2012), software for image acquisition (Weis and Hagen 2020; Cheng et al. 2018; Mastronarde 2018), and maturation of subclassification (Scheres 2016) and other in silico “purification” methods powered by graphics processing unit (GPU) computing. These developments have revolutionized how cell and molecular biologists are working to understand important biochemical processes. Not surprisingly, the average number of 3DEM density maps reported in a single published paper has increased over time (Fig. 1B). Twenty years ago, publications describing 3DEM structural studies encompassed an average of 1.5 3DEM density maps deposited into EMDB. Today, that metric has increased to an average of > 4 deposited 3DEM density maps per primary publication. 3D characterization of the flexible pantograph-like motion of transcribing RNA polymerase coupled to the translating ribosome involved deposition of 24 3DEM density maps to EMDB and deposition to the PDB of the same number of structures (i.e., atomic coordinates) (Wang et al. 2020).

Coinciding with exponential growth in the number of 3DEM structures in PDB, the average resolution of 3DEM structures archived in the PDB has improved dramatically. Since 2013, the average resolution of 3DEM structures in PDB has steadily improved from being slightly worse than 14 Å to better than 4 Å (Fig. 1C). Moreover, during this same time period, 43 PDB structures with resolution better than 2 Å have been deposited to PDB. As the resolution revolution continues, macromolecular crystallography (MX) software developers are increasingly crossing the “methodology barrier” to improve high-resolution 3DEM density maps even further (e.g., (Terwilliger et al. 2020)). As of mid-2022, the PDB archive housed > 7600 3DEM structures determined at near-atomic resolution (2–4 Å), with most recent depositions falling within the 3–4 Å resolution range (Fig. 1D). At this resolution, individual β strands and bulky amino acid residue sidechains are well resolved, both of which are essential for building atomic coordinate structure models into 3DEM density maps. Reaching 2–3 Å resolution is becoming somewhat routine for 3DEM structures deposited to PDB. The level of detail present in accompanying 3DEM density maps often permits accurate definition of the atomic coordinates without prior structural knowledge of individual macromolecular constituents from previously determined experimental structures or computed structure models (i.e., either from PDB or from AlphaFold 2 (Jumper et al. 2021), RoseTTAFold (Baek et al. 2021)).

3DEM structure determination beyond ~ 2 Å resolution appears unlikely to become routine. *Bona fide* atomic resolution (better than ~ 1.5 Å), wherein the positions of individual non-hydrogen atoms are discernable as isolated peaks in 3DEM density maps, has only been achieved to date for one biological specimen of exceptional stability and 3D structural homogeneity (human apoferritin), and the best-to-date being a ~ 1.15 Å resolution structure (PDB ID 7a6a (Yip et al. 2020)). Most samples of biological macromolecules embedded in vitreous ice may not have extremely well-ordered structures. It is remarkable that the average resolution of PDB MX structures plateaued at ~ 2.0 Å in ~ 1990 (S.K. Burley et al. 2022a), which probably reflects inherent both limitations in our ability to prepare well-ordered crystals of biological material and structural heterogeneity of many proteins found in nature.

As both quantity and quality of 3DEM structures in the PDB archive increase, it is gratifying to see that the boundaries of the method continue to be pushed (Fig. 1E). Since 2013, there has been a steady growth in 3DEM PDB structures wherein ligands have been modeled, which is a promising trend for the biopharmaceutical industry. In many cases, 3DEM can resolve structures of challenging/impossible-to-crystallize, high-value targets (e.g., integral membrane proteins (Robertson et al. 2022), see Area of Focus No. 3) for use in structure-guided drug discovery. Another interesting development of late is that 3DEM methods can now resolve sugars covalently bound to extracellular proteins. For example, PDB archive holdings of 3DEM structures of the highly glycosylated SARS-CoV-2 spike protein exceed ~ 1000 (see Area of Focus No. 4). Some of these data and earlier PDB structures of SARS-CoV spike proteins have informed design of vaccines (Goodsell and Burley 2022) and development of monoclonal antibodies to combat the COVID-19 pandemic (Gilliland et al. 2012; Chiu and Gilliland 2016). Equally impressive is the fact that previously encountered technical limitations of cryo-EM methods with respect to size (e.g., smaller < 200 kDa macromolecules) and complexity (e.g., distinct molecular entities $\ge$ 10) have been largely overcome. Finally, the future of cryo-ET combined with sub-tomogram averaging is looking very bright (see Area of Focus No. 5). As of mid-2022, the highest resolution cryo-ET structure in the PDB archive was PDB ID 7bzt (3.3 Å RuBisCO visualized within native *Halothiobacillus neapolitanus* carboxysomes (Cui et al. 2020)).

## “Resolution Revolution” areas of focus

Notwithstanding the milestones described above and the impressive metrics illustrated in Fig. 1, the impact of the resolution revolution can only be fully appreciated by delving into the structural biology literature. Six representative areas of focus are presented below, beginning with icosahedral viruses that served as “pioneer” samples in development of 3DEM methods and culminating with integrative or hybrid methods (I/HM) structural studies of complex macromolecular machines. These structures are so large and/or complicated that their determination necessitated combining 3DEM with complementary biophysical measurements. In many cases, I/HM structure determination was buttressed by structural information archived in the PDB and computed structure models coming from comparative protein structure modeling or artificial intelligence/machine learning methods.

## Area of Focus No. 1: 60-fold symmetric icosahedral viruses

The largest class of biological assemblies with regular non-crystallographic symmetry in the PDB archive is icosahedral viruses (as of mid-2022, count > 1100 structures). Throughout the 1980s and early-to-mid 1990s, such structures were determined exclusively using MX. In the late 1990s, however, the PDB began to receive depositions of atomic coordinates for icosahedral viruses obtained by fitting previously determined structures (already archived in the PDB) into low-resolution (20–30 Å) 3DEM density maps. Subsequently, vitrified icosahedral virus particles proved to be ideal specimens for 3DEM reconstruction software development, because the presence of 60 identical asymmetric units facilitated rapid and accurate orientation determination (Kaelber et al. 2017). The first 3DEM icosahedral virus structure made publicly available in the archive PDB was that of *Spiroplasma* virus in 1999 (PDB ID 1kvp (Chipman et al. 1998)). It was quickly followed by several 3DEM structures of viruses bound to cellular receptors or antibody fragments (e.g., rhinovirus: PDB ID 1d3i (Kolatkar et al. 1999) and1d3e (Kolatkar et al. 1999); poliovirus: PDB ID 1dgi (He et al. 2000); Foot and Mouth Disease virus: PDB ID 1qgc (Hewat et al. 1997)). Other early exemplars provided first demonstrations that high symmetry combined with 3DEM reconstruction could yield higher resolution structures, including Semliki Forest virus at 9 Å resolution (PDB ID 1dyl (Mancini et al. 2000)), and bacteriophages HK97 and PRD1, both determined at 12 Å resolution (PDB IDs 1if0 (Conway et al. 2001) and 1hb5 (Martin et al. 2001)).

All of the early 3DEM structures deposited to PDB were based on density maps reconstructed from single-particle images laboriously recorded and manually processed using photographic film. In the mid-2000s, charge-coupled device (CCD) detectors enabled automated 3DEM data collection (e.g., (Potter et al. 1999; Zhang et al. 2009; Mastronarde 2005)). Many virus-focused laboratories did not, however, embrace this new technology, primarily because CCD detectors were limited in terms of size and electron-detection sensitivity. Direct electron detectors were the key technology development that enabled rapid growth in numbers and increased resolution for icosahedral virus structures. Imaging virus particles in “movie mode” allows individual frames to be aligned, substantially reducing blurring from particle motion on the cryogenic sample stage that occurs during electron beam exposure (Campbell et al. 2012).

During the mid-2000’s, the wwPDB undertook the task of remediating virus structures with high non-crystallographic symmetry (Lawson et al. 2008). Three major issues were addressed, including (i) missing or erroneous sets of transformation operations, (ii) inconsistency in coordinate-frame representations, and (iii) overly complex instructions for building the virus assembly. A simplified uniform notation was adopted, yielding completely machine-readable instructions for building full virus assemblies in all icosahedral virus structures going forward.

As of mid-2022, 3DEM methods have been used to determine ~ 70% (~ 760) of all icosahedral virus structures archived in the PDB. Over the past 3 years, more than 100 new structures of icosahedral viruses have been publicly released by the PDB annually, with an average resolution of ~ 3.5 Å. The best resolved structure in this class is that of an adeno-associated virus, a gene therapy viral vector candidate, determined at 1.56 Å resolution (PDB ID 7kfr (Xie et al. 2020)). The “heaviest” icosahedral virus structure available in the PDB is that of Faustovirus (PDB ID 5j7v (Klose et al. 2016), Fig. 2). The Faustovirus is a 240-nm-diameter double-stranded DNA virus that infects amoebae. It consists of 5,340,600 amino acid residues weighing in at ~ 594,000 KDa. On the public health front, 3DEM structures of icosahedral viruses are informing our understanding of important viral pathogens. For example, atomic coordinates of Dengue, West Nile, and Zika flaviviruses have enabled mapping sites of glycosylation, amino-acid sequence variation, and neutralizing antibody binding and vaccine design (Goodsell and Burley 2022; Hasan et al. 2018).

**Fig. 2:**
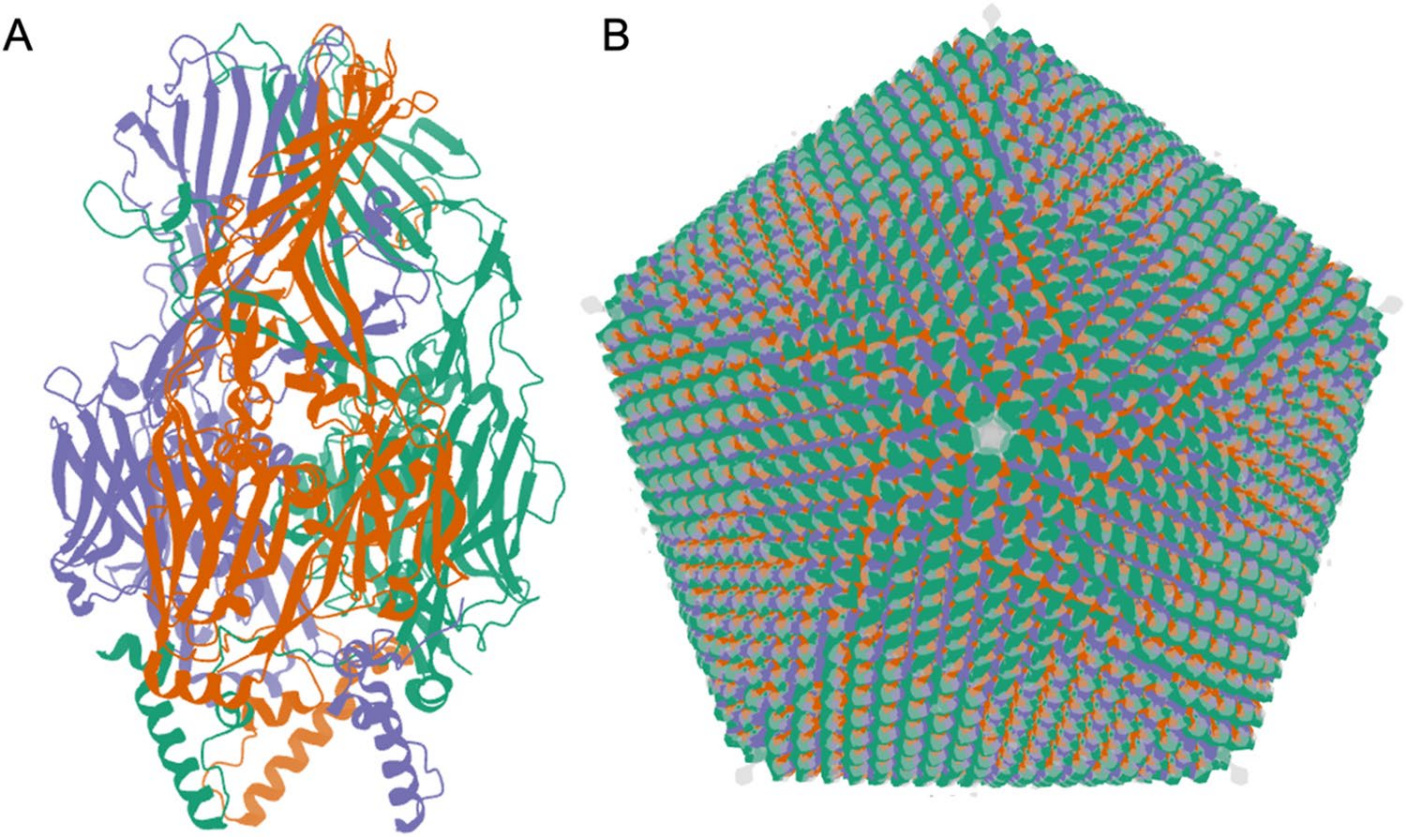
Faustovirus (Klose et al. 2016). **A**. 3DEM atomic coordinates in the PDB (entry PDB ID 5j7v) consist of the capsid protein trimer illustrated in ribbon representation. **B** Atomic coordinates for the full icosahedral capsid are generated by applying 2760 transformation matrices to the trimer atomic coordinates. The superimposed 15 Å resolution 3DEM density map (entry EMD-8144, in semi-transparent grey) reveals additional spike-like features for which atomic coordinates are not available extending from each fivefold vertex. Images generated using Mol* (Sehnal et al. 2021)

While icosahedral viruses are of substantial biological and biomedical importance in their own right, they continue to serve as real-world samples with which to improve 3DEM structure determination methodologies. These viruses are, as a rule, not perfectly icosahedral, although deviations from 60-fold symmetry are typically not observed until relatively large datasets and sophisticated approaches to image processing are employed (Kaelber et al. 2017). For example, newer methods have allowed visualization of the entire *Levivirus* genome asymmetrically packaged inside a capsid shell, which itself has icosahedral symmetry (Gorzelnik et al. 2016; Koning et al. 2016). Figure 1F documents that within the structural virology community, reliance on icosahedral averaging during structure determination declined markedly in 2016. This shift cannot be explained solely by increased emphasis on less symmetric viruses; it is even seen for members of the family *Picornaviridae*—a prototypically icosahedral virus. Moreover, intrinsic and induced asymmetries in viral capsids may become apparent when symmetry-breaking or symmetry-free reconstructions are used during structure determination. In one dramatic example, the structure of a Coxsackie virus capsid bound to nanodisc-embedded receptor exhibited receptor-induced asymmetry due to deformation of the ostensibly icosahedral capsid (PDB ID 3jd7 (Lee et al. 2016)). Looking to the future of structural virology, we can expect to learn a great deal more about dynamic, transient, and local conformational heterogeneity and flexibility that would otherwise remain obscured if we were limited to using MX with non-crystallographic symmetry restraints or 3DEM with icosahedral averaging for virus structure determination.

## Area of Focus No. 2: asymmetric ribosomal subunits and ribosomes

Ribosomes are responsible for messenger RNA template-directed protein synthesis in all living cells. They are also the targets of numerous antibiotics (Wilson 2014; Lin et al. 2018). The ribosome is a very large (2.5 MDa or more), highly intricate molecular machine comprising 40 or more distinct polypeptide chains apportioned between large and small subunits, plus 3 to 4 ribosomal RNA chains (totaling 1000s of RNA nucleotides). This picture is further complicated by the fact that ribosomes also form transient complexes with messenger RNAs (mRNAs), transfer RNAs (tRNAs), and numerous additional components responsible for regulation of translation throughout the processes of initiation, elongation, and termination. As structural studies of translation have evolved, they have been extended beyond the ribosome alone to capture mechanistic views of the ribonucleoprotein machine as the central player in many of the discrete biochemical steps occurring during protein synthesis, including translocation along mRNA, binding of tRNAs to their A, P, and E binding sites, and interactions with regulatory proteins, such as elongation factor-G or EF-G (Carbone et al. 2021; Noller et al. 2017).

Understanding mRNA translation at the atomic level became a reality in 2000 with determination of ribosomal subunit structures using MX: PDB IDs 1ffk (Ban et al. 2000), 1fka (Schluenzen et al. 2000), and 1fjg (Carter et al. 2000). Leaders of the research groups responsible for these landmark structures (Ada Yonath, Venki Ramakrishnan, and Thomas E. Steitz) shared the 2009 Nobel Prize in Chemistry. As of mid-2022, the PDB archive housed approximately 1600 structures identified as ribosomes. Of these, nearly 1000 were determined using 3DEM, versus ~ 600 determined using MX. 3DEM has become the method of choice for studying ribosome structure and function. Benefitting from technical advances described above, 3DEM structures of ribosomes are being determined at better than 2.0 Å resolution (e.g., PDB ID 7k00 (Watson et al. 2020)), and the average resolution of 3DEM ribosome structures archived in the PDB from the beginning of 2018 onwards is ~ 3.6 Å (versu*s* an average of ~ 3.1 Å for PDB MX structures of ribosome from the same period). 3DEM ribosome structures in PDB come from all of the kingdoms of life: ~ 31% eukaryotic, ~ 63% eubacterial, and ~ 6% archaebacterial.

**Fig. 3:**
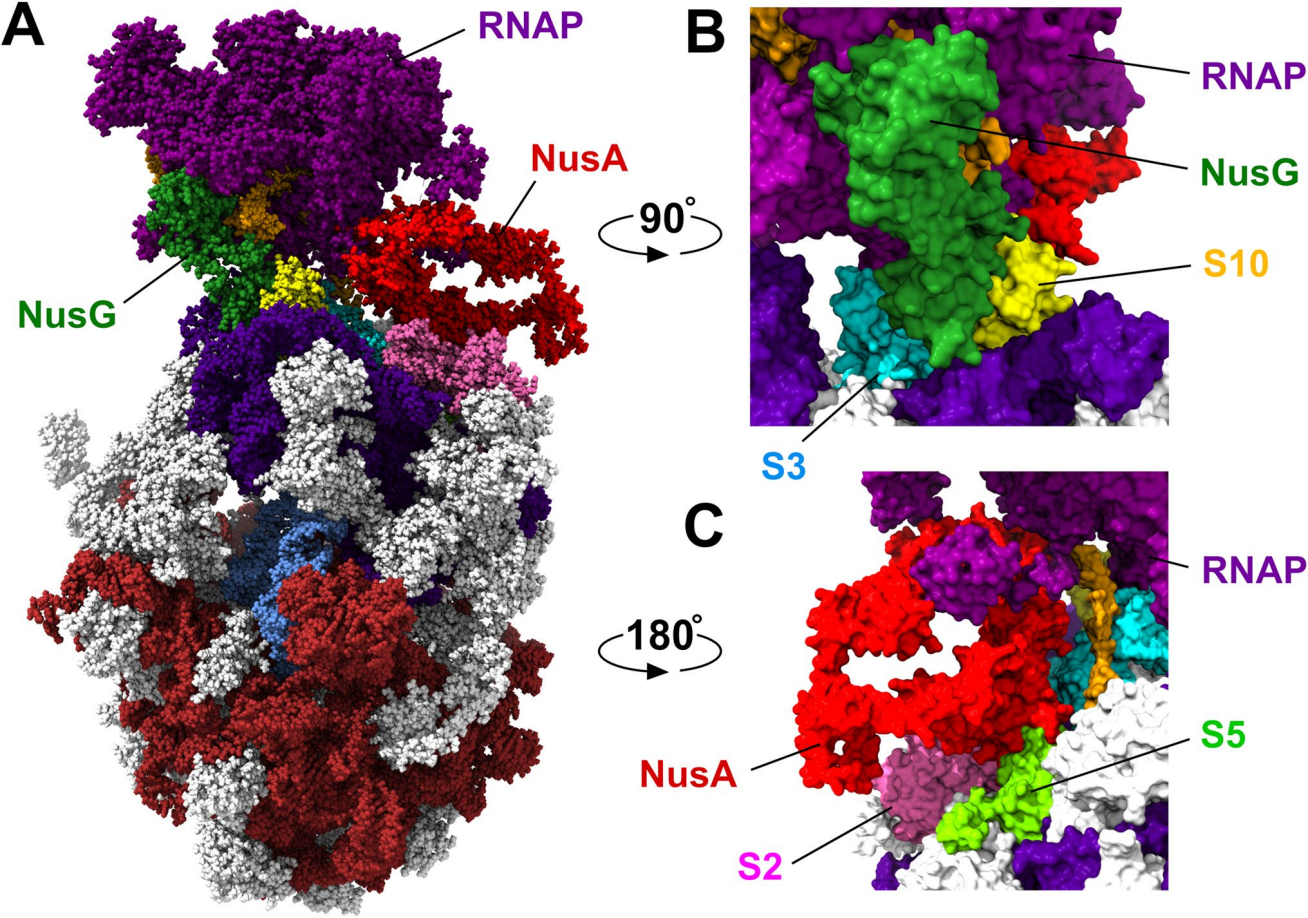
**A** *E. coli* transcription-translation complex (Wang et al. 2020) TTC-B2 (PDB ID 6x 7f), color coding: RNAP-purple, DNA-orange; ribosomal RNAs: large subunit-brown, small subunit-indigo; ribosomal proteins-grey (also see below); tRNA-blue; and transcription elongation factors NusG-dark green and NusA-red. The mRNA transcript is not visible in this representation. **B** Interaction of NusG (dark green) with RNAP (purple) and ribosomal proteins S3 (cyan) and S10 (yellow). **C** Interaction of NusA (red) with ribosomal proteins S2 (pink) and S5 (light green). Images generated using ChimeraX (Pettersen et al. 2021)

Illustrative of the advantages offered by 3DEM for functional studies is the recent work of Wang et al. (2020) studying transcription-translation coupling in bacteria. In prokaryotes, a transcription-translation complex (TTC) performs transcription of mRNA from DNA by RNA polymerase (RNAP) whilst that same mRNA transcript is being translated a ribosome yielding the polypeptide chain encoded by the gene. Formation of a TTC involves physical contact between RNAP and the ribosome. Previously published work demonstrated that coupling can be mediated by transcription elongation factors, such as NusG (Burmann et al. 2010) and NusA (Strauss et al. 2016). Wang et al. assembled *E. coli* TTCs comprising the DNA, RNAP, mRNA, ribosome, and tRNAs, both in the presence and absence of NusG and NusA. Their 3DEM structural studies of 24 distinct DNA, protein, RNA complexes detected four distinct TTC classes. One such class, TTC-B, observed in the presence of mild detergent with 8, 9, or 10 mRNA codons separating the RNAP and ribosome active sites, was identified as the functional complex (Fig. 3). Taking advantage of the fact that single-particle cryo-EM methods are not unduly compromised by sample heterogeneity or the presence of multiple conformational and/or configurational states present in electron images obtained from a single sample (Frank 2017), Wang et al. were able to subdivide TTC-B into subclasses (TTC-B1, TTC-B2, and TTC-B3) in which RNAP is differentially rotated relative to NusA and the ribosome. In all three TTC-B subclasses, NusG acts as a molecular bridge, binding to RNAP and bringing both itself and RNAP into contact with ribosomal proteins S10 and S3, respectively, in the 30S subunit. NusA in turn acts as a bridge, binding to RNAP and ribosomal proteins S2 and S5, while making no contacts with NusG. Uncovering of the structural bases of transcription-translation coupling by three research groups working independently highlights the convergence of single-particle cryo-EM of biochemically defined molecular species (Webster et al. 2020; Wang et al. 2020) with cryo-ET for in situ analyses (O’Reilly et al. 2020).

## Area of Focus No. 3: 3DEM studies of integral membrane proteins using single-particle methods

Membrane proteins represent nearly 60% of currently validated drug targets. Historically, determination of membrane protein structures posed significant technical challenges related to the complex lipid environment surrounding and stabilizing the protein. Bottlenecks in sample preparation for high-resolution structure determination by MX or 3DEM included sample extraction from biological membranes and solubilization and transfer into lipid vesicles in a manner that preserved both structure and function. Resulting samples were often aggregated and/or highly heterogeneous, rendering them unsuitable for crystallization or cryo-EM single-particle imaging. Recent breakthroughs in sample preparation largely overcame such challenges, making near atomic resolution structure determination of integral membrane proteins appear somewhat routine.

The first integral membrane protein structure deposited to the PDB was that of bacteriorhodopsin, determined using EC at 3.5 Å resolution by Henderson and coworkers (PDB ID 1brd (Henderson et al. 1990)). Subsequent discovery of detergents with low critical micellar concentration permitted extraction and transfer of individual membrane proteins from native membranes into lipid vesicles. This early approach was fraught with difficulties owing to variation in lipid vesicle size, complicating specimen vitrification, particle picking, classification, and alignment, and ultimately 3DEM structure determination. The first successful application of single-particle cryo-EM methods to studying the structure of an integral membrane protein yielded 3DEM density maps of the BK potassium channel at 17–20 Å resolution (EMD-5114 and EMD-5121 (Wang and Sigworth 2009)). Introduction of protein-lipid nanodiscs has enabled custom design of homogenous populations of lipid vesicle carriers for integral membrane proteins. Membrane scaffold protein (or MSP) nanodiscs (cube-biotech.com) are among the most popular at present. They consist of two copies of the amphipathic membrane scaffold protein composed of repeated $\alpha$-helix-forming segments. Two MSPs together form adjacent “belts” surrounding the aliphatic tails on the periphery of the disc-like lipid bilayer, thereby stabilizing the high-density lipoprotein-like assembly. Variations in the number of $\alpha$-helical repeats allow for control of nanodisc diameter, governing the size of membrane proteins that it can receive. The thickness of the lipid bilayer can also be controlled using homogeneous preparations of lipids with acyl chains of differing lengths. The composition of the nanodisc can be further optimized by including signaling phosphoinositides or cholesterol to create lipid raft-like environments.

Direct electron detectors (DEDs) were first used for structural studies of integral membrane proteins in 2013, yielding a 3.4 Å resolution structure of a mammalian TRP channel, TRPV1 (PDB ID 3j5q (Cao et al. 2013)). As of mid-2022, PDB holdings included 10,001 membrane protein structures (6591 determined by MX, 3370 by 3DEM, and 40 by EC). Approximately 99% of the 3DEM structures now archived in PDB relied on the use of DEDs. At the time of writing, the highest resolution 3DEM PDB structure of a membrane protein is that of a human gamma-aminobutyric acid receptor, the GABA(A)R-$\beta$ 3 homopentamer bound to histamine and megabody Mb25 (Nakane et al. 2020) determined at an overall resolution of 1.73 Å (as judged by the Fourier Shell Coefficient or FSC = 0.143 sigma criterion, see below). Locally, the resolution of the 3DEM density map ranges between ~ 1.6 Å and ~ 2.3 Å. As of mid-2022, the largest membrane protein-containing structure in the PDB was that of the 7546 kDa mitochondrial ATP synthase hexamer from *Toxoplasma gondii* (PDB ID 6tml (Muhleip et al. 2021)), which was determined at 4.8 Å resolution. With improving sample preparation methods, improved instrumentation, and advances in structure determination software many more exciting new 3DEM structures of integral membrane proteins will be deposited into the PDB in the coming years.

**Fig. 4:**
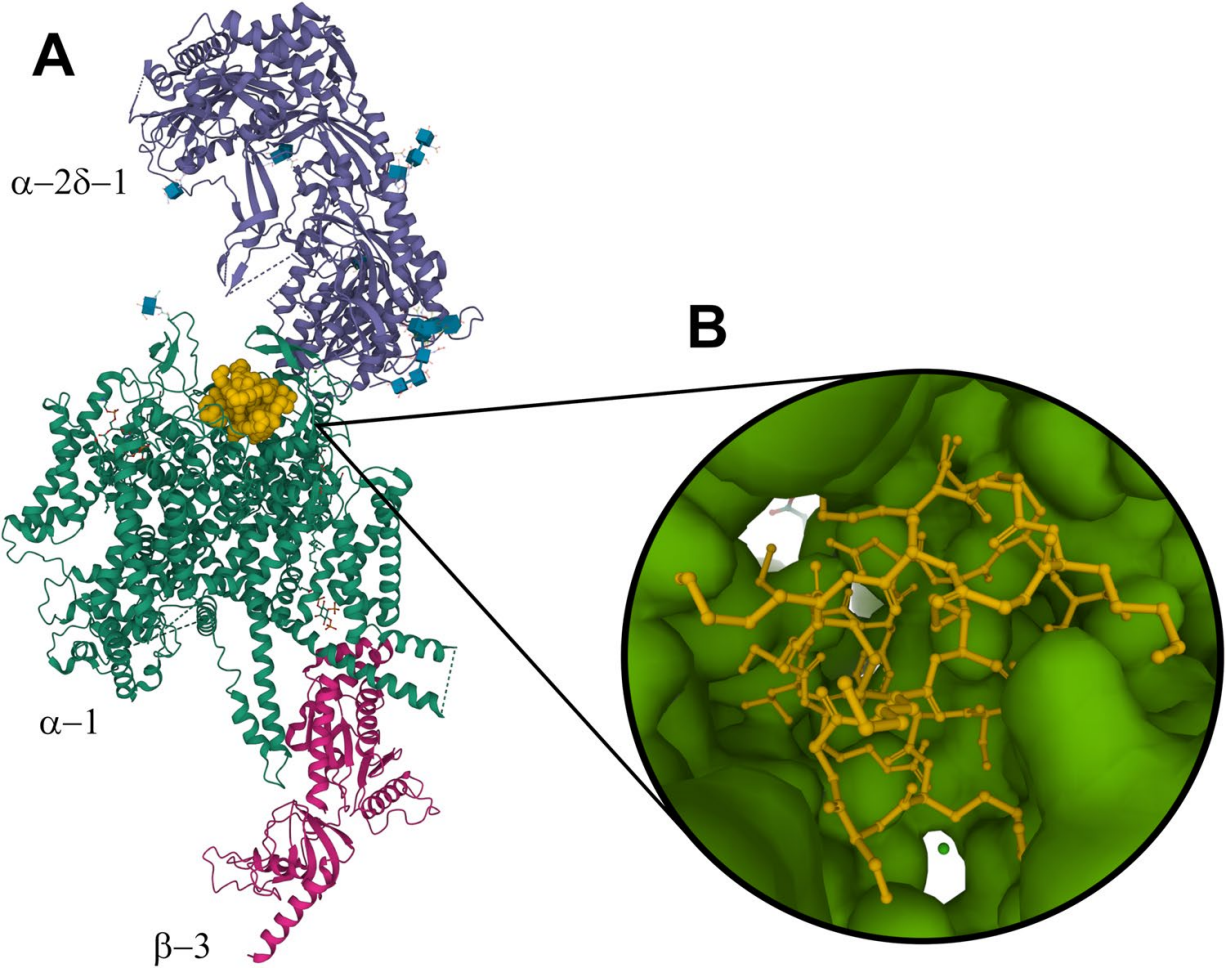
**A** Mol* ribbon representation of the 3DEM structure of the human Ca_v_2.2 bound to ziconotide (PDB ID 7mix (Gao et al. 2021). Color coding: ziconotide-orange (space-filling representation); $\alpha$-2 $\delta$-1 subunit-purple; $\alpha$-1 subunit-green; $\beta$-3 subunit-red. Glycosyl groups covalently bound to the $\alpha$-2 $\delta$-1 and $\alpha$-1 subunits are displayed as blue cubes with atomic stick figures using the GlycanBuilder representation described in (Shao et al. 2021). **B** Rotated Mol* closeup representation of the interaction of ziconotide (ball-and-stick) with $\alpha$-1 (surface)

Of particular importance in structure-based drug discovery is the advent of 3DEM studies of small-molecules bound to membrane proteins that represent potential drug discovery targets. Figure 4, for example, illustrates the structure of human Ca_v_2.2, a neuronal-type voltage-gated calcium channel bound to ziconotide, a United States (US) Food and Drug Administration (FDA)-approved drug prescribed for treating intractable pain. Yan and co-workers used a single-particle cryo-EM to reveal how the drug blocks the ion-conducting pore of Ca_v_2.2 (Gao et al. 2021). Ziconotide is a biologic, a neurotoxic peptide derived from the cone snail *Conus magus*, comprising 25 amino acids with three disulfide bridges. It is administered via intrathecal injection into the spinal canal. Understanding the mode of action of this biologic agent at the atomic level could enable structure-guided discovery of orally bioavailable small-molecule organic compounds for effective management of chronic pain without exposing patients to the risk of opiate addiction.

## Area of Focus No. 4: SARS-CoV-2 spike proteins

Coronaviruses were named for their appearance in negative stain electron micrographs showing a “corona,” which we now know to be due to the presence of spike proteins arrayed on the surfaces of individual virions. These spikes are transmembrane glycoproteins responsible for mediating viral entry into host cells and inducing neutralizing immune responses. They are also the antigens presented to the immune system by the two mRNA vaccines now in common use to combat the COVID-19 pandemic (Goodsell and Burley 2022).

**Fig. 5:**
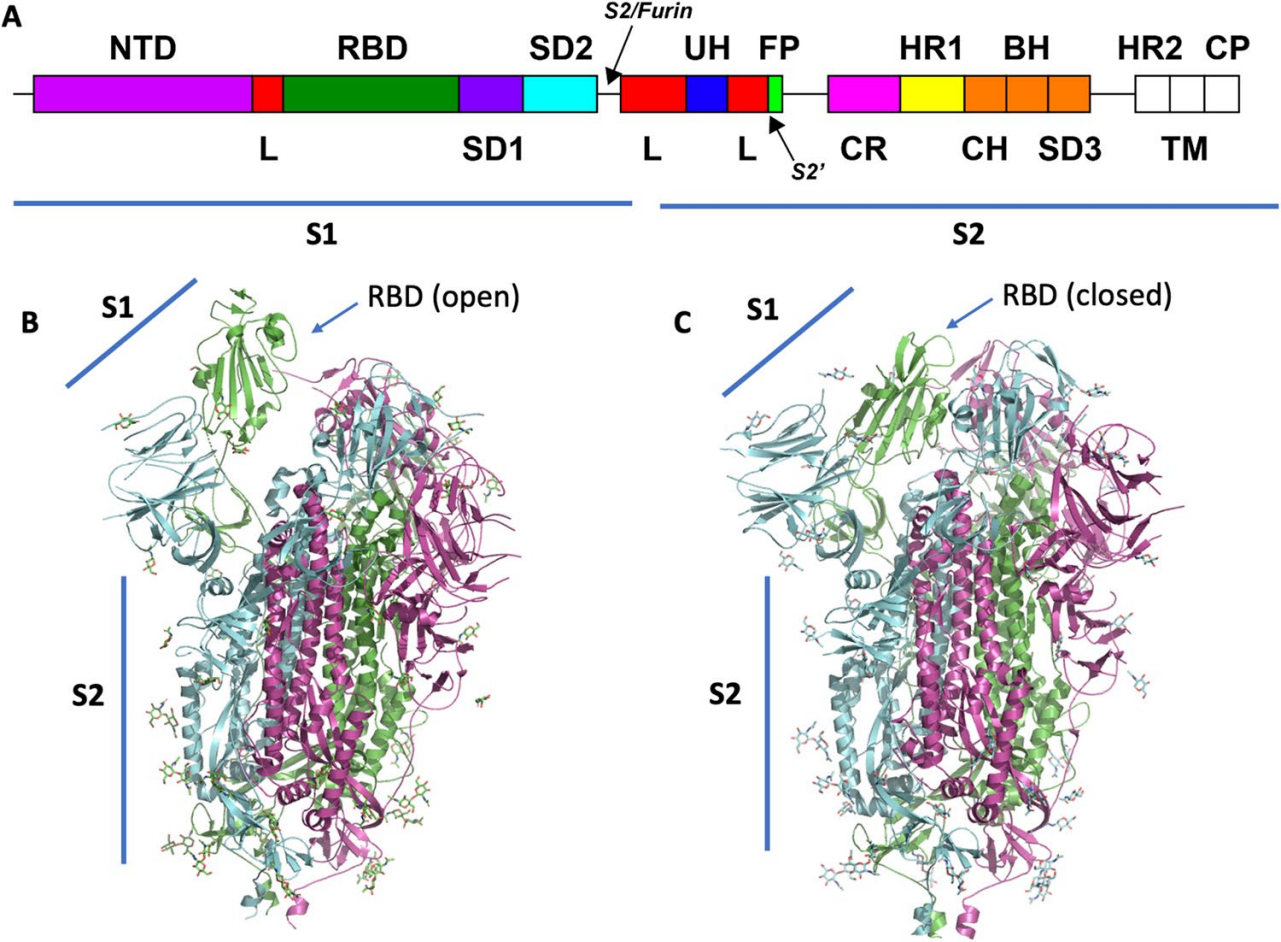
**A** Schematic view of SARS-CoV-2 spike protein sequence showing arrangement of polypeptide chain segments S1 and S2 and various domains. Proteolytic cleavage sites are indicated with arrows. **B** Mol* ribbon representation of the one-up-two-down RBD conformation of the spike protein (PDB ID 6vsb (Wrapp et al. 2020)). **C** Mol* ribbon representation of the all-down RBD conformation observed in PDB ID 6vxx (Walls et al. 2020). Individual trimers are color-coded magenta, green, and cyan, respectively. Covalently bound glycosyl groups are depicted as atomic stick figures

In SARS-CoV-2, spike proteins found on the surfaces of mature virions occur as clover-shaped homotrimers, with three receptor-binding S1 segments sitting atop a trimeric membrane-fusion S2 segment stalk (Wrapp et al. 2020; Walls et al. 2020). Each S1 segment contains a receptor-binding domain (RBD) and an N-terminal domain (NTD) (Fig. 5A). During viral entry, the RBD binds to one or more of its host receptors (e.g., angiotensin-converting enzyme 2, ACE2), mediating virion attachment (Shang et al. 2020). Short amino acid sequence motifs at the S1/S2 inter-segment boundary and/or S2′ site are cleaved specifically by host proteases (Xia et al. 2020). ACE2 binding and proteolytic cleavage together trigger S1 to dissociate (Benton et al. 2020) from the trimer. Then S2 undergoes a large structural change leading to fusion of the viral and host cell membranes, thereby allowing the genetic material of the virus to enter the host cell (Cai et al. 2020). 3DEM structures of SARS-CoV-2 spike protein hold the key to understanding the complex process of cell entry and evolution of immune evasion by variants of concern identified by the World Health Organization.

Technical advances described above enabled extremely rapid determination of spike protein structures within months of the onset of the COVID-19 pandemic and identification of the novel coronavirus SARS-CoV-2 as the causative infectious agent. The first PDB structure of the spike protein was determined at ~ 3.5 Å resolution via 3DEM by McLellan and colleagues (PDB ID 6vsb; Fig. 5B (Wrapp et al. 2020)) and made publicly available in late February 2020. This structure of the pre-fusion spike protein showed one RBD within the trimer adopted the “standing up” conformation ready to engage ACE2 on the cell surface, whereas the other two RBDs of the trimer adopted the “lying down” position, which sequesters the receptor-binding motif (one up-two down conformation). Within weeks, Zhuo and colleagues and Veesler and colleagues released the structures of ACE2-RBD complex (PDB ID 6m17; resolution 2.9 Å (Yan et al. 2020)) and the spike protein in the three-RBD-down state (PDB ID 6vxx; resolution 2.9 Å (Walls et al. 2020); Fig. 5C), respectively, both determined using single-particle cryo-EM. By July 2020, Chen and colleagues had determined the structure of the post-fusion conformation of the spike (PDB ID 6xra; resolution 3.0 Å (Cai et al. 2020)). Detailed comparison of pre- and post-fusion structures revealed conformational changes mediating fusion of viral and host cell membranes. The highest resolution structure of the prefusion spike as of mid-2022 was determined by Veesler and colleagues, who included NTD-binding antibodies to stabilize the structure of the trimer (PDB ID 7lxy; resolution 2.20 Å (McCallum et al. 2021)). Notably, Subramaniam and colleagues determined the structures of the Delta and Kappa variants of the spike protein at relatively high resolution (PDB IDs 7tey, 7tf3; resolution 2.25 Å (Saville et al. 2022)). Brunger and colleagues determined the structure of an intermediate conformation in the fusion process (PDB ID 7rzq, resolution 2.09 Å (Yang et al. 2022)). As of mid-2022, there were > 700 structures of the SARS-CoV-2 spike protein in the PDB. Many of these PDB IDs include bound monoclonal antibodies or other designed proteins, enabling mechanistic characterization of therapeutic agents and establishing opportunities discovery and development of new or modified biologics to combat emerging variants of concern (Hunt et al. 2022).

## Area of Focus No. 5: cryo-electron tomography with sub-tomogram averaging

Over the past two decades, cryo-ET has emerged as an exciting new approach to structure determination of biological specimens in their native, hydrated states. Single-particle cryo-EM requires purification of biomolecules of interest, which may disrupt molecular interactions, modify environment-specific conformations, or even eliminate biologically relevant contextual information. Sample preparation for cryo-ET, in contrast, does not require isolating biological molecules from their native cellular or subcellular milieux. This in situ method provides unique opportunities for visualizing macromolecular machines exhibiting compositional and/or conformational heterogeneity, membrane-associated complexes, or high-order arrangements, all within complex native environments.

Once a 3D tomogram has been recorded by tilting the sample stage within the electron microscope and imaging projections at multiple angles, objects of interest in the electron micrographs can be extracted digitally and further processed by sub-tomogram averaging. To produce a high-resolution 3D cryo-ET density map, sub-tomograms of the same object are iteratively aligned and averaged to increase signal-to-noise ratio and address missing wedge artifacts intrinsic to cryo-ET due to mechanical tilt limitations of the sample stage. Because cryo-ET data collection is lower throughput and more computationally demanding than single-particle cryo-EM, sub-tomogram averaging is a less popular method of 3DEM structure determination. As of mid-2022, there were only 229 cryo-ET structures in the PDB (~ 2% of all 3DEM archival holdings).

The very first cryo-ET structures deposited into the PDB were those of rigor cross-bridges in insect flight muscle (PDB IDs 1m8q, 1mvw, 1o18, 1o19, 1o1a, 1o1b, 1o1c, 1o1d, 1o1e, 1o1f, 1o1g (Chen et al. 2002)) and cadherins visualized within desmosomes (PDB IDs 1q55, 1q5a, 1q5b, and 1q5c (He et al. 2003)). In these pioneering studies, tomograms of chemically preserved samples were used to generate density envelopes for positioning of known PDB structures. Despite being low resolution, both studies delineated domain organization and interactions between subunits. For the insect flight muscle case, atomic-level PDB structures of actin (PDB ID 1atn (Kabsch et al. 1990)) and myosin sub-fragment (PDB ID 2mys (Rayment et al. 1993)) positioned as rigid bodies by real-space refinement yielded new interaction information. These pioneering studies propelled cryo-ET towards the forefront of structural biology and laid the groundwork for integrative, multiscale structural biology combining data from complementary techniques to reveal more a complete “picture” of proteins visualized in their native environment.

Cryo-ET has come a long way since the early 2000s. Over the past two decades, technical advances in cryo-electron microscopy, DEDs, and state-of-the-art software for automated data acquisition and image processing (to name a few, M (Tegunov et al. 2021), emClarity (Himes and Zhang 2018), EMAN2 (Chen et al. 2019)) have led to significant improvements in 3D structure determination via sub-tomogram averaging. In 2020, structures of capsid domain (CA) in lentivirus equine infectious anemia virus (EIAV) immature Gag lattices assembled at two different pH conditions were resolved at sub-4 Å resolution via sub-tomogram averaging (PDB IDs 6t61, 6t64, and 6t63 (Dick et al. 2020)). In 2021, 3DEM structures of the 70S ribosome in *Mycoplasma pneumoniae* cells (PDB IDs 7ood and 7p6z (Xue et al. 2021)) were determined at 3.4 Å resolution. These PDB structures highlight the rapid growth and maturation of cryo-ET as a mainstream tool for structure determination at near-atomic resolutions and its potential for revealing complex structures involved in dynamic processes within organelles and cells.

Most early cryo-ET studies were focused on enriched organelles, membrane fractions, or small prokaryotic cells, because electron beams of transmission electron microscopes are unable to penetrate thicker eukaryotic cells. Sample milling using focused ion beams at cryogenic temperatures (cryo-FIB) was developed to prepare thinned samples without unduly damaging the specimen. This method can produce compression-free, electron-transparent lamellae, substantially extending the range of biological samples that can be investigated by cryo-ET. As of mid-2022, the highest resolution cryo-ET structure in the PDB employing cryo-FIB milling (3.3 Å) was that of RuBisCO visualized within native *Halothiobacillus neapolitanus* carboxysomes (PDB ID 7bzt (Cui et al. 2020)).

**Fig. 6:**
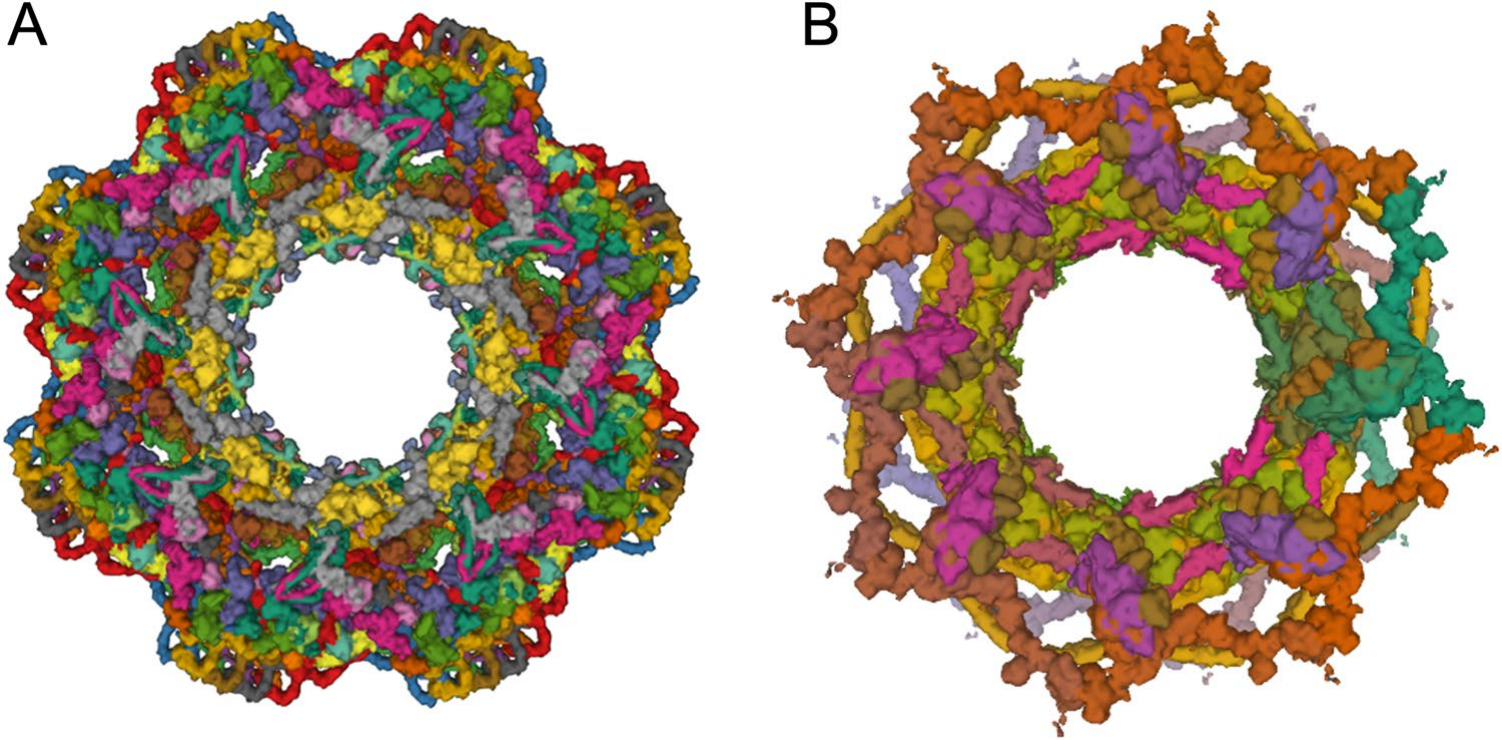
**A** Cryo-ET structure of the eightfold symmetric human NPC in its constricted state determined at 12 Å resolution (PDB ID 7r5k (Mosalaganti et al. 2022)). **B** Integrative structure of yeast NPC with eight spokes (PDBDEV_00000012) determined using the *Integrative Modeling Platform* (Kim et al. 2018). Images generated using Mol*

Immediate-term prospects for cryo-FIB milling followed by cryo-ET combined with sub-tomogram averaging brightened considerably with the advent of AlphaFold 2 (Jumper et al. 2021) and RoseTTAFold (Baek et al. 2021). For example, computed structure models of human nuclear pore complex (NPC) proteins from AlphaFold DB (Varadi et al. 2022) were combined with cellular cryo-ET and molecular dynamics simulations to generate composite 3DEM density maps of the human NPC in both dilated and constricted conformations (PDB IDs 7r5k, 7tbl, 7tbm, 7tbj, 7tbk, and 7tbi (Mosalaganti et al. 2022). Figure 6A illustrates a projection view of a nominal 12 Å resolution structure of the human NPC in its constricted state. The human NPC consists of ~ 1000 distinct polypeptide chains, nearly double that of the yeast NPC discussed in Area of Focus No. 6 and illustrated in Fig. 6B.

With advances in sample preparation, data processing, and protein structure prediction, cryo-ET has become a versatile tool for capturing the structural dynamics and spatial arrangements of macromolecular machines within cellular landscapes. Ongoing efforts in optimizing correlative light-electron microscopy will facilitate identification of macromolecular assemblies present in what are often crowded cellular environments. Additionally, use of artificial intelligence and machine learning-based algorithms to accurately annotate individual molecular assemblies within cells has already played a significant role in structure interpretation and bias-free, high-throughput sub-tomogram extraction for downstream sub-tomogram analysis (Chen et al. 2017; Che et al. 2018; Moebel et al. 2021). These exciting developments will open new opportunities for studying a broader range of biological systems in situ at unprecedented resolution.

## Area of Focus No. 6: PDB-Dev integrative structures largely reliant on 3DEM

PDB-Dev (pdb-dev.wwpdb.org, (Vallat et al. 2018; Vallat et al. 2021; Burley et al. 2017)) is the wwPDB prototype repository for archiving integrative structures of large macromolecular assemblies. Integrative or hybrid methods structure determination involves measuring data with complementary experimental methods and combining this information with previously determined 3D structures or computed structure models of individual components to assemble a set of spatial restraints. “Integrative structures” are then determined through an iterative computational process known as satisfaction of spatial restraints (Rout and Sali 2019). The resulting integrative structures may include individual atomic coordinates and/or coarse-grained representations. Experimental tools commonly used in integrative structure determinations include cryo-EM and cryo-ET, small-angle scattering (SAS), chemical crosslinking mass spectrometry (CX-MS), hydrogen–deuterium exchange mass spectrometry, Forster resonance energy transfer, and electron paramagnetic resonance spectroscopy.

In the face of growing interest in integrative structural biology, the wwPDB established an integrative/hybrid methods task force to identify and recommend how best to address challenges involved in archiving, validating, visualizing, and disseminating these structures (Sali et al. 2015; Berman et al. 2019). The first order of business was creation of data representations for different kinds of experimental restraints. To support PDB-Dev, the PDBx/mmCIF data representation (Westbrook et al. 2022; Westbrook et al. 2005; Fitzgerald et al. 2005; John D. Westbrook and Fitzgerald 2009) that underpins the PDB archive was extended to represent integrative structures and associated experimental information. In addition, new software tools, a data harvesting system, and a website for data delivery were built (Vallat et al. 2021, 2019). At the time of writing, work is underway to develop methods for validating integrative structures based on the recommendations of the task force (Berman et al. 2019).

PDB-Dev was launched in 2016 with three integrative structures determined using the *Integrative Modeling Platform* (Russel et al. 2012) (Nup87 (PDBDEV_00000001 (Shi et al. 2014)); Exosome (PDBDEV_00000002 (Shi et al. 2015)); and Mediator (PDBDEV_00000003 (Robinson et al. 2015)). As of mid-2022, PDB-Dev holdings encompassed nearly 100 publicly released structures (or entries), plus some fully processed entries to be released on publication. The inaugural PDB-Dev integrative structure based largely on 3DEM data is that of the Mediator complex (PDBDEV_00000003 (Robinson et al. 2015)). Currently, there are 20 structures in PDB-Dev (including 19 released structures and 1 on hold pending publication) largely based on 3DEM. Ten of these 20 3DEM-based PDB-Dev structures also used distance restraints measured using CX-MS.

In 2018, an integrative structure of the yeast NPC consisting of 552 polypeptide chains was determined with the *Integrative Modeling Platform* (Fig. 6B) at sub-nanometer precision (Kim et al. 2018). The corresponding PDB-Dev submission consists of three related entries: PDBDEV_00000010 (single spoke), PDBDEV_00000011 (3 spokes), and PDBDEV_00000012 (8 spokes). Experimental restraints for the yeast NPC were obtained from single-particle cryo-EM, 2D EM class averages, SAS, and CX-MS. 3D structures of individual NPC components were obtained from PDB, PDB-Dev, or generated via comparative protein structure modeling. The integrative structure of the yeast NPC can be sub-divided into the membrane ring, inner and outer rings, a cytoplasmic export platform, the nuclear basket and the disordered FG repeats that fill the pore. The integrative structure of the yeast NPC provided insights into underlying architectural principles, mechanisms of transport across the nuclear membrane, functional regulation, and assembly/disassembly processes and broadened our understanding of the evolutionary origins of NPCs (Akey et al. 2022; Petrovic et al. 2022; Bley et al. 2022; Zimmerli et al. 2021; Allegretti et al. 2020; Mosalaganti et al. 2018).

## 3DEM structure validation by the wwPDB

As is the case for MX and NMR, validation standards for 3DEM structures archived in the PDB are being developed collaboratively by the wwPDB and community experts. The inaugural wwPDB 3DEM Validation Task Force (VTF) Workshop (Henderson et al. 2012) provided initial recommendations, including use of FSC for objective assessment of 3DEM density map resolution (Rosenthal and Henderson 2003). The wwPDB 3DEM VTF also recommended development of new criteria for evaluation of 3DEM density maps and emphasized the importance of statistically rigorous assessment of the fit of atomic coordinates to 3DEM density maps.

Based on the outcome of the 2011 Data Management Challenges in 3DEM Workshop (Patwardhan et al. 2012), new services for 3DEM data depositors were developed, including the EMPIAR archive (Iudin et al. 2016), standalone FSC and tilt-pair services (Patwardhan and Lawson 2016; Wasilewski and Rosenthal 2014), and Visual Analysis web pages (Abbott et al. 2018; Lagerstedt et al. 2013).

Following the onset of the 3DEM resolution revolution (Kuhlbrandt 2014), there was substantial growth in the number of moderate-to-high resolution 3DEM structures deposited to PDB, inducing community experts to update their recommendations for archiving and validating 3DEM structures and experimental 3DEM density maps and related metadata. The EMDataResource (EMDR) has organized highly influential community challenges to assess ongoing improvements in both structure determination software and 3DEM structure and density map validation (Lawson et al. 2020, 2016). In 2016, EMDR sponsored two separate challenges (Heymann et al. 2018). One evaluated density map generation while the other evaluated atomic coordinates-to-map fitting, concluding at the 2017 EMDR Joint Challenges Workshop (Lawson and Chiu 2018). At the Workshop, it became painfully apparent that different practitioners can arrive at very different 3DEM density map resolution estimates from exactly the same data. Community-wide adoption of a standardized method for estimating 3DEM density map resolution was identified as being critical for the well-being of the field. Thereafter, deposition of experimental half-maps became mandatory, enabling consistent, objective assessment of resolution using the FSC = 0.143 criterion by the wwPDB OneDep software system for every 3DEM structure deposited to PDB and every 3DEM density map deposited to EMDB.

At the time of writing, the FSC = 0.143 sigma criterion had been broadly adopted within the 3DEM community. Alternative cutoffs such as the FSC = 0.5 sigma criterion, and the half-bit criterion have been derived from first principles and/or empirically (van Heel and Schatz 2005). For example, FSC = 0.5 corresponds to the resolution at which the signal-to-noise ratio is unity (Baldwin and Lyumkis 2020). Although reducing resolution to a single, consistent number with FSC = 0.143 has clear practical value, no single number can fully encapsulate resolution when it comes to 3DEM density maps. Resolution may vary as a function of direction (Baldwin and Lyumkis 2020) or spatial location within the sample (Nakane et al. 2018), and frequency-dependent fall off in the signal-to-noise ratio can differ among density maps of similar nominal resolution. The most common reason for resolution to vary by direction is non-uniform sampling that occurs when the biological specimen assumes a preferred orientation on the planar EM grid, typically because of adherence to the air–water interface or substrate-water interface at the edge of the layer of cryopreserved liquid (Baldwin and Lyumkis 2020). Approaches to conveying more information concerning density map resolution include: depositing a full FSC curve for the entire density map, using a tool such as 3DFSC (Tan et al. 2017) to assess directional anisotropy, or using tools to assess local variations in resolution, such as ResMap (Kucukelbir et al. 2014) or MonoDir (Vilas et al. 2020).

Following the 2019 Cryo-EM model challenge, the 2019 Model Metrics Workshop was convened to assess findings and make recommendations (Lawson et al. 2021). Key workshop recommendations were as follows: (a) archives can independently estimate resolution by FSC from deposited unmasked, minimally filtered half-maps to eliminate differences observed in depositor-derived resolution estimates; (b) EMRinger (Barad et al. 2015) scores reflect map and model quality for certain amino acid residue sidechains; (c) *Q*-scores (Pintilie et al. 2020) reflect both local 3DEM density map quality and fit of atomic coordinates to these maps; (d) CaBLAM (Williams et al. 2018) and Molprobity (Chen et al. 2010) cis-peptide detection can be used to evaluate protein backbone conformation; (e) 3DEM map density-based cross-correlation scores (Farabella et al. 2015; Afonine et al. 2018; Joseph et al. 2017; Vasishtan and Topf 2011) and atom inclusion (Lagerstedt et al. 2013) can be used to evaluate atomic coordinate model-to-map fit; (f) *Z*-scores can be used instead of raw scores for several metrics evaluated; and (g) use of refined atomic displacement parameters (ADPs) or B-factors for 3DEM should be investigated.

At the time of writing, wwPDB validation reports for 3DEM structures include: (a) assessment of model geometry similar to that used for all MX and NMR structures (ClashScores, Ramachandran outliers, sidechain outliers, nucleic acid polymer backbone outliers); (b) orthogonal projections of map and map-model overlays; (c) half-map FSC plot based on mandatory half-maps collected at deposition; (d) voxel-value distribution and volume-estimation graph; (e) evaluation of map-model fit via atom-inclusion plot and residue inclusion analysis; and (f) finer evaluation of resolvability and atomic coordinate model-to-map fit, incorporating both overall and per residue *Q*-scores (Pintilie et al. 2020). EMDB also provides 3DEM density map and structure quality assessments on its website, including *Q*-scores (Z. Wang et al. 2022).

**Fig. 7:**
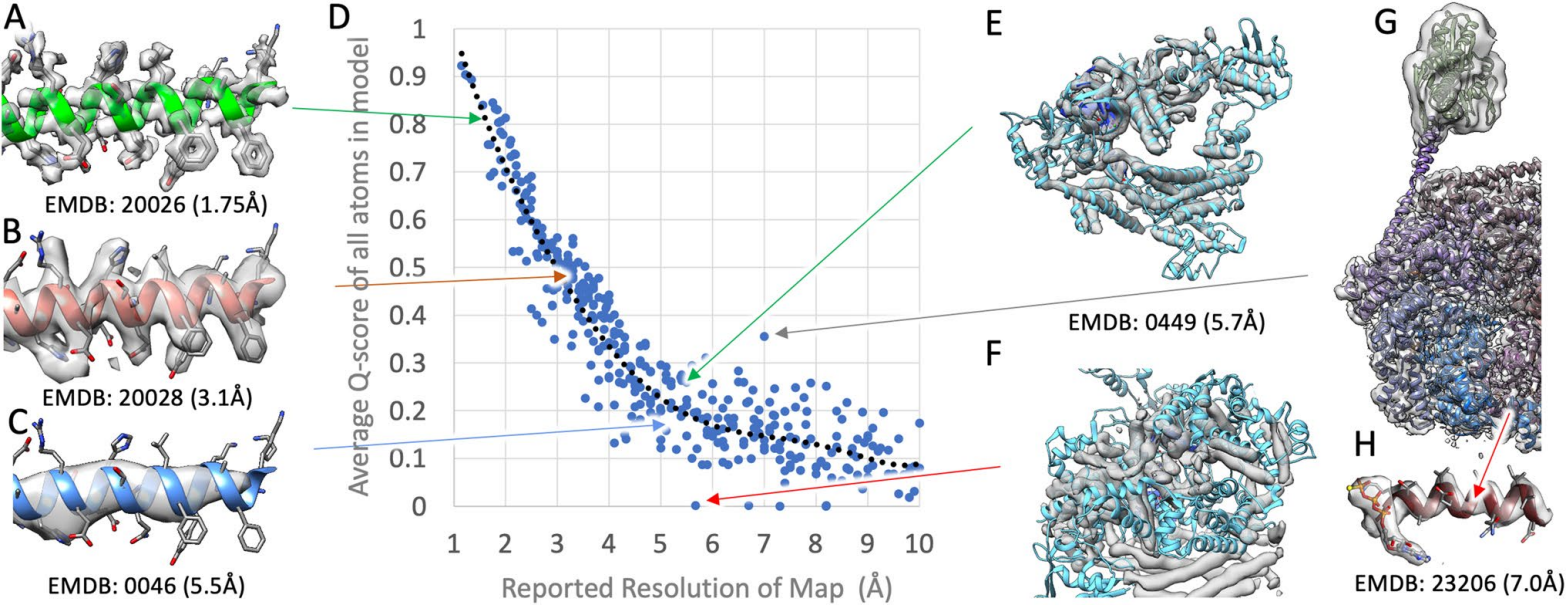
Extracted portions of 3DEM density maps and corresponding atomic models shown in Panels **A**–**C**, with arrows indicating their overall *Q*-score values in the plot of *Q*-score versus reported density map resolution plot (Panel **D**). The plot was based on 374 EMDB density maps released between 2018 and 2021, randomly chosen such that resolution is evenly distributed between ~ 1 and ~ 10 Å. Panels **E** and **F** show for PDB ID 6nme/EMD-0449 (H. Zhang et al. 2019) that even at a lower resolution (~ 5.5 Å), an atypical *Q*-score value near zero can indicate an improper global fit of the atomic coordinates to the 3DEM density map. Panels **G** and **H** show PDB ID 7l6n/EMD-23206 (Yin et al. 2021) for which the reported resolution is ~ 7.0 Å. Actual resolvability is higher as indicated by the *Q*-score of ~ 0.36 (versus the value of ~ 0.16 expected at the reported resolution)

Now that *Q*-scores are being used more widely to assess 3DEM density maps archived in EMDB, experience has confirmed their utility as measures of both resolvability and atomic coordinate model-to-map fit. Figure 7 illustrates how resolvability of maps at different resolutions is reflected in overall *Q*-score values (Fig. 7A–C). Even at lower resolution, an atypical Q-score value near zero can signify an improper model-to-map fit, as shown by Fig. 7E (re-aligned corrected fit, *Q*-score ~ 0.27) and Fig. 7F (mis-aligned incorrect fit, *Q-*score ~ 0). Figure 7G and H illustrate a 3DEM structure and related experimental density map for which the reported resolution is ~ 7.0 Å, although overall resolvability is higher as indicated by a *Q*-score ~ 0.36, which is well above the expected *Q*-score at that resolution. Comparison of the 3DEM density map and the atomic model confirms that while some parts of the structure are not well resolved, much of the density map has resolvability approaching ~ 4 Å resolution (e.g., helix pitch is clearly visible in Fig. 7H). Overall Q-score values can, therefore, be used for independent assessment of depositor reported resolution (which may not reflect the value computed by the wwPDB using the FSC = 0.143 sigma criterion where 3DEM density half maps are available), and as an aid to identifying incorrectly fitted atomic coordinates.

**Fig. 8:**
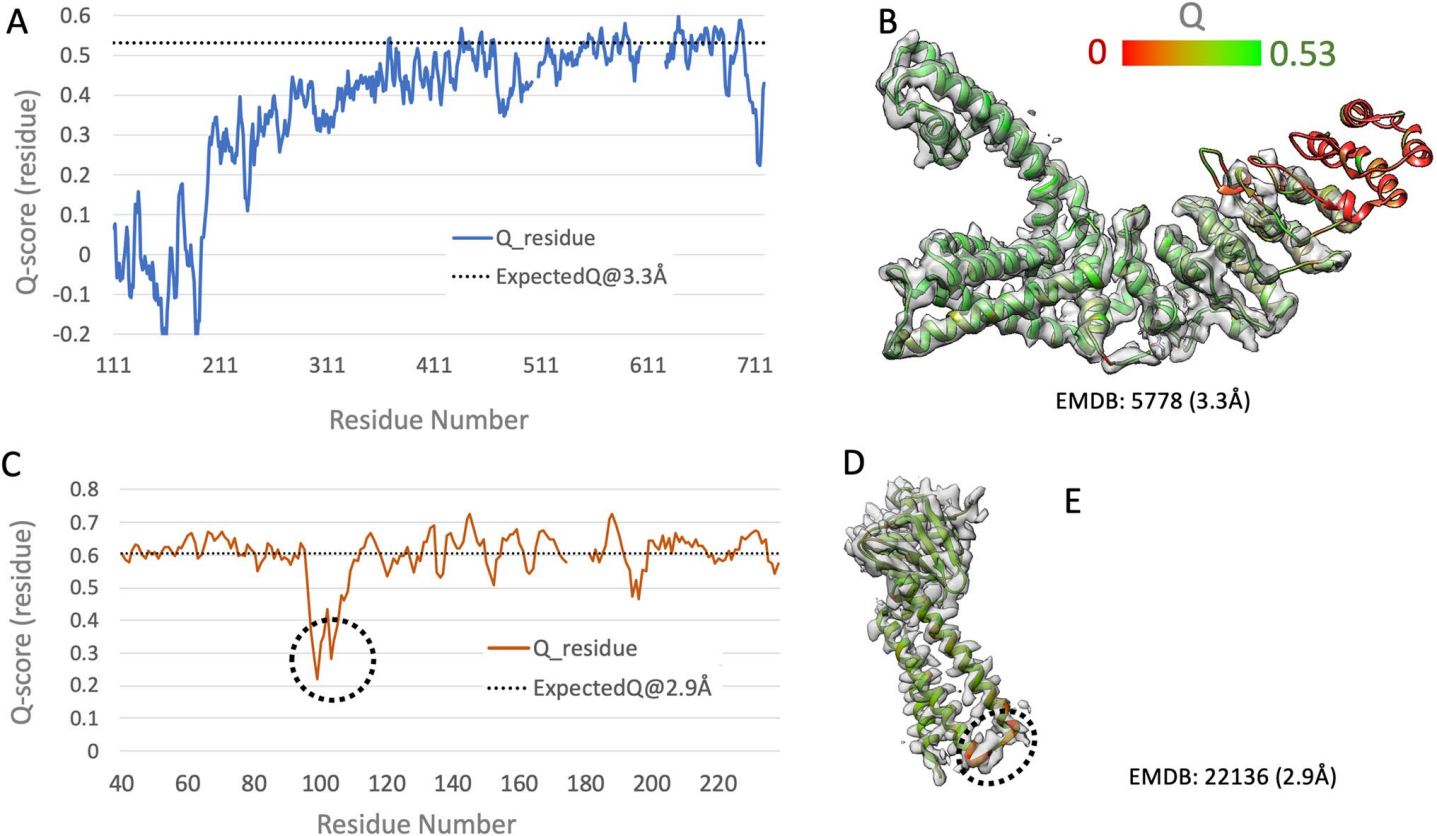
Per-residue *Q*-scores can be used to assess resolvability (panels **A** and **B**) and identify opportunities to improve atomic coordinate model-to-map fit (panels **C**–**E**). Panels **A** and **C** show per-residue *Q*-scores versus residue number for PDB ID 3j5p/EMD-5778 (resolution 3.3 Å (Liao et al. 2013)) and PDB ID 6xdc/EMD-22136 (resolution 2.9 Å (Kern et al. 2021)), respectively. Average per-residue *Q*-score for reported resolution of the 3DEM density map resolution is shown as a horizontal dotted line, based on the dotted line fit to overall *Q*-scores *versus* reported density map resolution (Fig. 7D). Panels **A** and **B** illustrate how per-residue *Q*-scores falling below expected average values can be used to identify segments of the polypeptide chain that are not well resolved (given the reported density map resolution). Panels **C**–**E** illustrate how per-residue *Q*-scores falling below expected average values can be used to identify segments of the polypeptide chain wherein the atomic coordinate model-to-map fit is not consistent

The relationship between *Q*-score and resolution depicted in Fig. 7D can be also used together with per-residue *Q*-score plots and 3DEM density map/atomic coordinate model visualization to identify regions of the structure wherein the density map is less well resolved than expected (Fig. 8A, B). Some samples studied using 3DEM are dynamic and conformationally heterogeneous under identical experimental conditions on the same EM grid. 3DEM density maps are typically less well resolved in such cases, particularly when averaging has been performed across large numbers of conformationally distinct particles. The FSC = 01.43 sigma criterion-based resolution of such 3DEM density maps may reflect the well-resolved portions of the sample. Fitted atomic coordinates can also be annotated with per-residue *Q*-scores to identify parts of structure that are less well resolved. Detailed comparison of 3DEM structures and related density maps, available respectively from PDB and EMDB, sometimes reveals that the atomic coordinates were not correctly fit to the experimental density map. Such cases typically exhibit lower-than-expected *Q*-scores, as shown in Fig. 8C–E for PDB ID 6xdc/EMD-22136. The plot of per residue *Q*-score versus residue number (Fig. 8C) reveals a deep minimum in the vicinity of residue number 100, wherein the computed *Q*-score is significantly lower than the value expected at 2.9 Å resolution (horizontal dotted line). Visual inspection of the 3DEM density map overlaid with the atomic model (Fig. 8D, E) reveals inconsistencies between the atomic coordinates and the experimental density map for the loop segment of the polypeptide chain connecting two long $\alpha$-helices. In contrast, the atomic coordinate model-to-map fit for the pair of $\alpha$-helices is entirely consistent (Fig. 7D). The two use cases presented in Fig. 8 exemplify the value of careful scrutiny of per-residue Q-scores and the fit of atomic coordinates to the density map prior to deposition of 3DEM structures to PDB and density maps to EMDB using the wwPDB OneDep software system. PDB depositors are strongly encouraged to use the wwPDB standalone validation system (https://validate.wwpdb.org) both during structure determination and before depositing any 3DEM structure to the PDB or 3DEM density maps to EMDB via OneDep.

## Future perspectives

Looking ahead, the prospects of single-particle cryo-EM, cryo-ET, and microED as 3D biostructure detemination methods appear very bright. There is every reason to believe that the number of 3DEM structures and density maps deposited annually to the PDB and EMDB, respectively, will continue to increase year-on-year for some time. It also appears likely that the number of single-particle cryo-EM studies yielding only 3DEM density maps with no accompanying atomic-level PDB structure(s) will decline as instrumentation, methodology, and software continue to improve (and journal editors and referees “raise the bar” for publication). When, if ever, the number of annual PDB depositions of cryo-ET structures coming from sub-tomogram averaging will begin to rival the productivity of single-particle cryo-EM practitioners is not clear. But individual in situ structures could well have a much larger impact on our understanding of the inner workings of organelles and cells than many single-particle cryo-EM structures. Whatever the merits of cryo-ET versus single-particle cryo-EM structures, open access to computed structure models of essentially any protein can only serve to accelerate progress for both techniques with enormous potential benefits accruing to basic and applied research in fundamental biology, biomedicine, bioengineering, biotechnology, and energy sciences.

At the time of writing, price inflation had returned to many economies around the world, as “too many dollars chasing too few goods” made each dollar less valuable. Structural biology as a discipline and the “market for 3D structures of biological macromolecules,” in contrast, do not appear to obey the laws of macroeconomics. Between the beginning of 2013 and the end of 2021, the PDB more than doubled in terms of number of structures archived (growing from 86,184 to 185,472). In late 2022 or early 2023, the total number of structures stored in the PDB will almost certainly exceed 200,000. But structural biologists and the structures they deposit to the PDB are not perceived as declining in value. If anything, 3D biostructures being published in 2022 are viewed as more valuable, as are structural biologists (particularly those with major accomplishments in cryo-EM or cryo-ET).

3D structures of biological macromolecules are not, of course, the same as dollar bills. Relentless growth in the PDB has been accompanied by increased complexity of newly deposited 3D biostructures. Average PDB structure size (i.e., assessed by the number of amino acid and nucleotide residues comprising the sample) has increased (Burley et al. 2022a). The same is true for the average number of distinct polymer chains/PDB ID and the average number of small-molecule ligands/PDB ID (Burley et al. 2022a). Simply put, while the number of structures archived in the PDB has grown, newly deposited structures are becoming more “interesting” and, hence, more valuable to the research community. Some of the growth in structure size and structure complexity can be attributed to the success of the 3DEM “Resolution Revolution.” Structural biologists are no longer hostages to what can be crystallized or rendered adequately soluble in an NMR tube. They are putting ever larger, ever more complex macromolecular machines onto EM grids, and, in favorable cases, “seeing” at the atomic level how the individual protein and nucleic acid chains are arranged, and how individual macromolecules recognize one another and how they bind to small-molecule ligands, such as enzyme co-factors, substrates, inhibitors, investigational agents, and US FDA-approved drugs.

In due course, however, today’s electron microscopists are likely to find, as previous generations of structural biologists did with MX and NMR, that much of the “low-hanging fruit” has been picked. They will turn, as some have already done, to integrative or hybrid methods to tackle challenging systems wherein NMR, MX, or 3DEM are not by themselves sufficient for structure determination. Instead, these three mainstays of structural biology and the PDB will come to be viewed as important tools that must often be combined with other biophysical measurement techniques to determine 3D structures of larger and more complex experimental systems. This trend is already evidenced by the growing number of protein crystallographers reinventing themselves as electron microscopists, driven by the conviction that in biology “function follows form.” Beginning with the structures of the DNA duplex (Watson and Crick 1953) and sperm whale myoglobin (Kendrew et al. 1960), generations of structuralists have shown that it can pay handsomely to study systems in 3D at the atomic level in order to understand biological phenomena and it can also pay to be nimble.
